# The role of peer-led mental health training in undergraduate medical education - a way forward?

**DOI:** 10.1192/j.eurpsy.2021.1247

**Published:** 2021-08-13

**Authors:** G. Cole

**Affiliations:** Medical School, University of Bristol, Bristol, United Kingdom

**Keywords:** Medical Education, peer-to-peer, training

## Abstract

**Introduction:**

Mental health is no doubt a topical conversation at medical school. We noted that whilst many students appreciated the power in talking openly about challenges faced, it was a topic many found hard to approach. In response, we have implemented a peer-led training programme at Bristol Medical School. The aim of the programme is to improve confidence and enable students to recognise and respond to their own, a peer or patient’s distress in a more proactive, supportive and overall effective way. It utilised peer-led, discussion based workshops during the first few months of medical school to achieve this.

**Objectives:**

To evaluate the role of peer-led mental health training in undergraduate medical education.

**Methods:**

The program was piloted in November 2019. T-tests compared 142 participating students’ baseline self-reported understanding and confidence and follow up, as measured on a likert scale (1-5). Qualitative feedback was also welcomed.

**Results:**

Students showed a significant improvement in their self-reported understanding (24%, P<0.05), confidence when supporting a peer (18%, P<0.05) and confidence if faced with a more acute situation (21%, P<0.05). Students expressed particular admiration for the fact that the session was peer led ‘as it emphasised the importance of mental health in…society’.

**Conclusions:**

This programme may be beneficial in creating a stronger community of doctors who are equipped with the confidence and ability to better care for themselves, their colleagues, and patients. Further evaluation is required to determine whether this reduces rate or severity of mental illness in participants or the broader student population.

